# Liquid biopsy in gynecological cancers: a translational framework from molecular insights to precision oncology and clinical practice

**DOI:** 10.1186/s13046-025-03371-1

**Published:** 2025-05-08

**Authors:** Canio Martinelli, Alfredo Ercoli, Giuseppe Vizzielli, Sharon Raffaella Burk, Maria Cuomo, Vrunda Satasiya, Housem Kacem, Simone Braccia, Giulio Mazzarotti, Irene Miriello, Manuela Nana Tchamou, Stefano Restaino, Martina Arcieri, Alice Poli, Veronica Tius, Silvana Parisi, Stefano Pergolizzi, Giuseppe Iatì, Chiara Conti Nibali, Cristina Pizzimenti, Ludovica Pepe, Antonio Ieni, Salvatore Cortellino, Antonio Giordano

**Affiliations:** 1 Sbarro Institute for Cancer Research and Molecular Medicine and Center of Biotechnology, College of Science and Technology, Temple University, 1900 N 12 St, Philadelphia, PA 19122 USA; 2 Department of Human Pathology of Adult and Childhood “Gaetano Barresi”, Unit of Obstetrics and Gynecology, University of Messina, Via Consolare Valeria 1, Messina, 98124 Italy; 3 Clinic of Obstetrics and Gynecology, Santa Maria Della Misericordia” University Hospital, Azienda Sanitaria Universitaria Friuli Centrale, Udine, Italy; 4 Department of Medical Biotechnology, University of Siena, Via Aldo Moro 2, Siena, 53100 Italy; 5 Department of Life, Health and Environmental Sciences, University of L’Aquila, L’Aquila, Italy; 6 Department of Pharmacy, School of Medicine, University of Naples Federico II, Via Domenico Montesano 49, Naples, 80131 Italy; 7 Radiation Oncology Unit, Department of Biomedical, Dental Science and Morphological and Functional Images, University of Messina, Messina, 98125 Italy; 8 Section of Pathological Anatomy, Department of Human Pathology of Adult and Evolutive Age “Gaetano Barresi”, G. Martino Hospital, Messina, 98125 Italy; 9 Clinical and Translational Oncology, Scuola Superiore Meridionale (SSM), Naples, Italy; 10 Laboratory of Molecular Oncology, Research Hospital, Campobasso, 86100 Italy; 11 SHRO Italia Foundation ETS, Candiolo, Turin, Italy

**Keywords:** Liquid biopsy, Gynecological cancer, Translational medicine, Clinical implementation, Precision oncology, Narrative review

## Abstract

Liquid biopsy offers a noninvasive method to identify and monitor tumor-derived biomarkers, including circulating tumor DNA (ctDNA), circulating tumor cells (CTCs), exosomes, microRNAs, and tumor-educated platelets, that provide real-time insights into the biological behavior of gynecological cancers. The detection of these markers has the potential to revolutionize cancer management by enabling earlier detection, providing novel data to personalize treatments, and predicting disease recurrence before clinical imaging can confirm progression, thereby also guiding complex clinical decision-making. However, because this new “omics” layer introduces additional complexity, it must be fully understood, from its biological rationale to technical development and clinical integration, to prevent confusion or misapplication. That is why, focusing on 14 critical fields of inquiry, our goal is to map the current state of liquid biopsy from bench to bedside while highlighting practical considerations for clinical integration. Each topic integrates recent advances in assay sensitivity, biomarker variability, and data interpretation, underscoring how standardized protocols and robust analytical methods are pivotal for reliable results. We then translate these findings into disease-specific insights, examining how liquid biopsy could refine early detection, minimal residual disease assessment, and therapy guidance in endometrial, cervical, and ovarian cancers. Although several FDA-approved assays and promising commercial tests illustrate the field’s rapid evolution, many translational hurdles remain, including the need for harmonized protocols, larger prospective clinical trials, and cost-effectiveness analyses. Crucially, our synthesis clarifies the pivotal role of interdisciplinary collaboration. Oncologists, laboratory scientists, and industry partners must align on standardized procedures and clinically relevant endpoints. Without such coordination, promising biomarkers may remain confined to research settings, limiting their practical benefit. Taken together, our review offers a translational view designed to contextualize liquid biopsy in gynecological oncology.

## Introduction

The era of precision oncology mandates a paradigm shift in diagnosing, monitoring, and treating gynecological malignancies. Over 1.3 million women in the United States, encompassing 862,875 endometrial, 295,748 cervical, and 238,484 ovarian cancer cases, highlight the urgent need to translate molecular insights into clinical practice [1]. In this context, liquid biopsy has emerged as a non-invasive diagnostic tool with significant promise, facilitating the detection of tumor-derived biomarkers [2–7]. Despite this promise, translating emerging molecular findings into routine gynecological oncology care remains challenging. Each step, from in vitro experiments to clinical validation, introduces distinct hurdles, as illustrated by Curry’s conceptual framework on translational science [8]. Moreover, the complexity of oncology requires approaches that do not overwhelm practitioners, making standardized, domain-agnostic strategies critical for practical implementation [9]. In gynecological oncology specifically, protocols must consider the molecular heterogeneity seen across endometrial, cervical, and ovarian cancers, all while remaining feasible in real-world settings. A truly translational perspective recognizes the bidirectional nature of research and clinical practice: clinicians require clear guidance on interpreting and applying molecular data, while scientists need insights from clinical realities to refine diagnostics and treatments [10, 11]. Understanding this interplay is paramount in gynecological oncology, where the nuances of tumor biology intersect with patient-centered care needs. This narrative review thus spotlights liquid biopsy as both a scientific breakthrough and a case study in effective translation. By examining its technical advances alongside real-world considerations, we aim to clarify how liquid biopsy can be harnessed to enhance gynecological cancer care.

## Methods

We adopted a narrative, translational approach to address 14 critical issues in liquid biopsy for gynecological oncology, guided by the PRISMA flowchart (Supplementary Document 1). Each issue was explored either via focused search strategies or an experience-based review (Table 1):

- Narrative Exploration: Foundational topics were identified through landmark studies and the authors’ expertise.
- Targeted Searches: Where specific data were needed, we used predefined keywords in PubMed, Embase, and Cochrane (2019–2024), limiting to English-language human studies of clear relevance.
- Screening: Retrieved articles were manually screened to exclude non-gynecological malignancies, case reports, editorials, or non-original data.

**Table 1:**
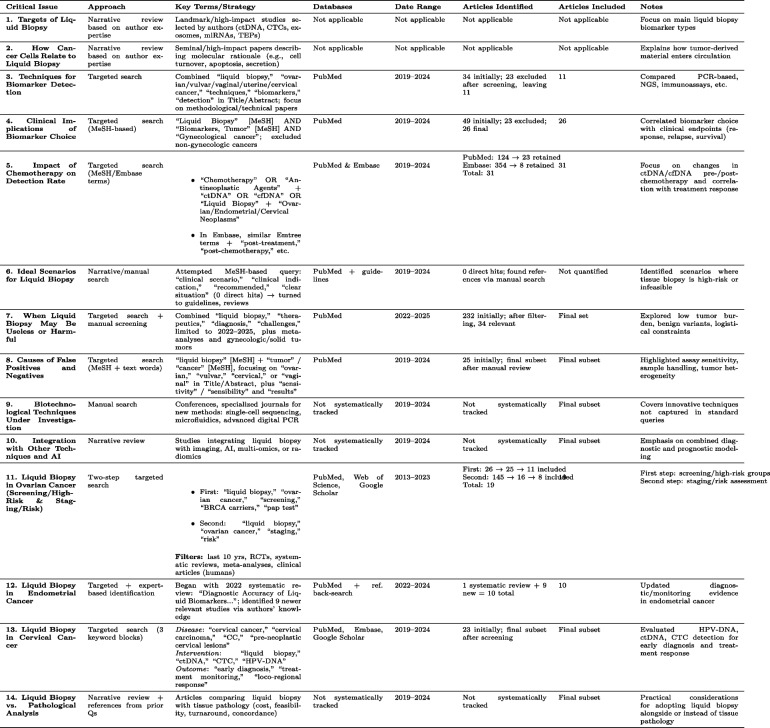
Summary of approaches, search strategies, and outcomes for 14 critical issue related to liquid biopsy in gynecological cancers

Overall, our goal was to integrate molecular rationale, technical considerations, and real-world clinical implications for each query.

### The targets of liquid biopsy

**Fig. 1:**
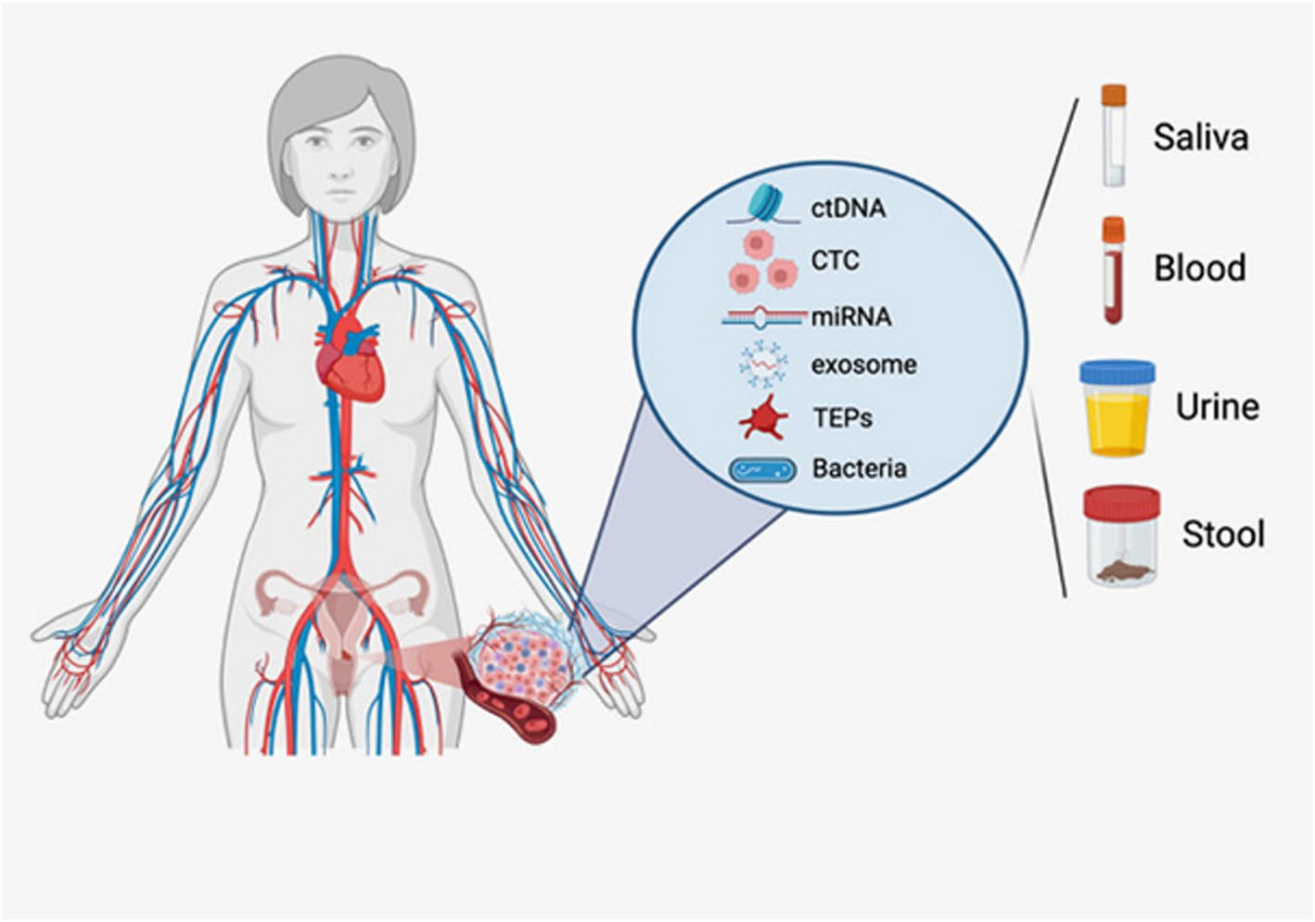
Graphic representation of liquid biopsy markers and sources for cancer detection. Liquid biopsy markers encompass circulating tumor cells, extracellular vesicles, tumor-derived microRNAs, and microbial cell-free DNA derived from the microbiota, which can be utilized to extrapolate cancer-associated microbiota signatures. These markers are released and detectable in various bodily fluids, including blood, urine, saliva, or stool (created with BioRender.com)

Liquid biopsy represents a significant advancement in cancer diagnostics by enabling noninvasive sampling of tumor-derived materials from blood, urine, ascitic fluid, pleural fluid, cerebrospinal fluid, sputum, saliva, and feces [2–7] (Table 2 and Fig. 1). The principal analytes include:

**Table 2:**
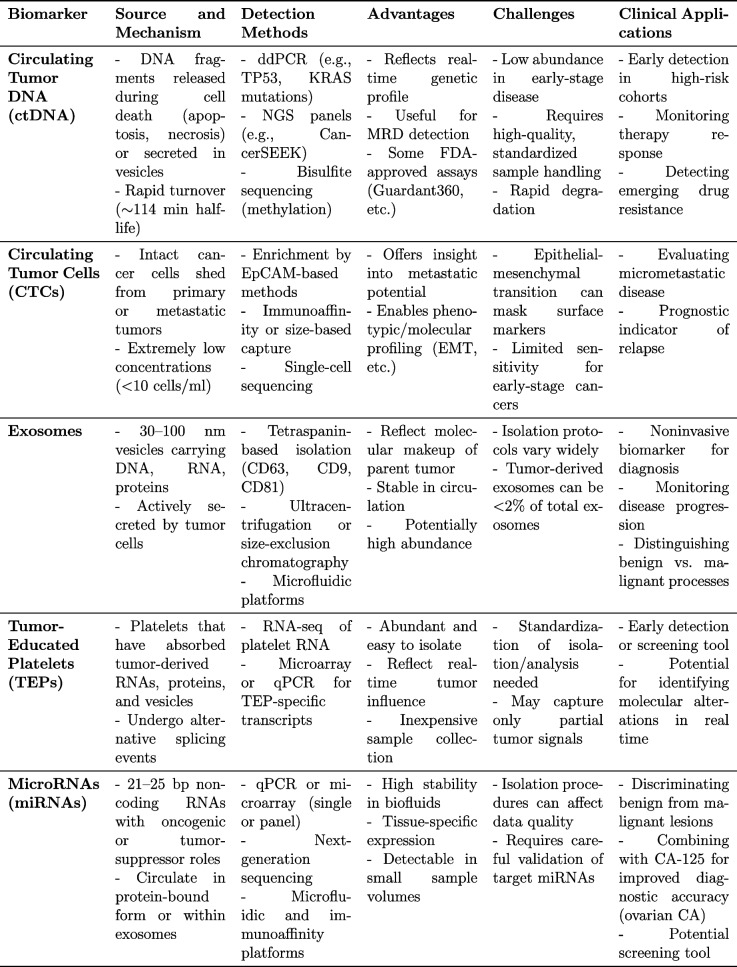
Summary of liquid biopsy biomarkers in gynecological cancers

- Cell-free DNA (cfDNA) and Circulating Tumor DNA (ctDNA)
  Cell-free DNA (cfDNA) refers to DNA fragments found in circulation, and its tumor-derived fraction is termed ctDNA. In healthy individuals, plasma cfDNA levels range from 65 to 877 ng/ml, while those in cancer patients often exceed 1000 ng/ml [12]. This tumor-derived fraction possesses genetic and epigenetic alterations and typically has a brief half-life of around 114 minutes [13]. Clearance primarily occurs via the reticuloendothelial system, with the liver accounting for 70–90%, spleen ~3%, kidneys ~4%, and the remainder undergoing enzymatic degradation [14, 15].
- Circulating Tumor Cells (CTCs)
  CTCs are intact cancer cells shed from primary or metastatic sites into the bloodstream, generally at very low concentrations (<10 cells/ml) [8]. Their half-life ranges from 1 to 2.4 hours, and they are commonly identified using epithelial markers such as epithelial cell adhesion molecule (EpCAM) or via distinct cellular traits [16].
- Tumor-Educated Platelets (TEPs)
  TEPs are platelets that have absorbed tumor-derived materials (e.g., mRNA, proteins, vesicles), undergoing characteristic changes in RNA and protein expression [17–20]. These alterations can distinguish them from normal platelets and offer insights into tumor biology.
- Exosomes
  Exosomes are 30–100 nm vesicles formed via endocytic pathways [21–25]. They carry DNA, RNA, miRNAs, and proteins, and can be isolated using tetraspanins like CD63, CD9, and CD81 [26]. Because they reflect the molecular makeup of their cells of origin, exosomes play crucial roles in tumor communication and metastasis.
- MicroRNAs (miRNAs)
  These 21–25 base-pair non-coding RNA molecules circulate either within vesicles or bound to proteins. They can act as either oncogenes or tumor suppressors, often displaying altered expression in cancer [27, 28]. They are attractive biomarkers for various gynecological malignancies.

### How cancer relates to liquid biopsy targets in gynecologic oncology

Although each liquid biopsy component ultimately reflects tumor presence, each biomarker enters circulation through distinct biological mechanisms:

- CfDNA enters the circulation via apoptosis, necrosis, and NETosis [29]. During apoptosis, DNA is fragmented by enzymes such as DNA fragmentation factor B (DFFB), DNASE1, and DNASE1L3 [30, 31], and may also be released under conditions of cellular stress or injury [32]. Furthermore, their altered chromatin structure, which is more “open” due to intensified transcription, renders DNA susceptible to nuclease-mediated fragmentation [33]. These unique fragmentation patterns mirror profound changes in nuclear organization and gene expression seen in malignant transformation [34–36], a field referred to as “fragmentomics.” In circulation, it is protected by binding to nucleosomes, argonauts, and lipoproteins (HDL, LDL), or by encapsulation in vesicles [37, 38]. Cancer cells can also actively secrete DNA through extracellular vesicles, reflecting their high metabolic activity [39].
- CTCs enter the bloodstream when cells detach from the primary tumor or metastases, reflecting key molecular alterations essential for malignancy. These include modified cell adhesion, heightened survival mechanisms, and resistance to anoikis (programmed cell death caused by detachment from the extracellular matrix). Their mere presence in peripheral blood points to the tumor’s invasive potential [16, 40]
- TEPs are platelets whose molecular profiles have been reshaped by close interaction with the tumor microenvironment. Cancer cells release soluble factors, such as RNA and proteins, which platelets absorb, triggering alternative splicing events. These modifications indicate both direct tumor influence and the body’s wider response to malignancy [17–20]. Although early-phase or “phase 0” studies in gynecological cancers have hinted at the strong potential of TEPs for cancer detection and monitoring, robust and up-to-date evidence specifically within gynecological malignancies remains limited [41, 42].
- Exosomes in cancer undergo changes in both quantity and composition because malignant cells frequently boost exosome production as part of survival and growth strategies, promoting cell-to-cell communication, remodeling the tumor microenvironment, facilitating invasive behavior, and contributing to drug resistance. Moreover, their cargo, mutated DNA, regulatory RNAs, and proteins, mirrors the parental tumor’s molecular signature [22–25].
- Abnormal miRNA profiles in cancer represent a breakdown of crucial regulatory pathways. Elevated or depleted miRNA expression can bolster the malignant phenotype. These small RNAs reach circulation via active vesicular secretion or cell death; they remain stable either by binding protective proteins or by encapsulation in vesicles. Their distinct expression signatures in patient plasma often correlate with tumor presence and progression [25, 27, 28].

### Techniques for biomarker detection

From a practical standpoint, having the option to analyze multiple biomarkers makes liquid biopsy especially appealing to clinicians. Nonetheless, standardizing protocols, managing costs, and accurately interpreting complex genomic data remain significant barriers to routine adoption. The principal methods include:

- DNA methylation represents one of the earliest and most stable cancer-associated alterations. It is typically assessed via bisulfite conversion, which distinguishes methylated from unmethylated cytosines [43]. Panels targeting genes such as RASSF1A, OPCML, and BRCA1 can achieve sensitivity and specificity as high as 91%, thereby increasing confidence in early malignancy detection [44]. More advanced methods, including high-resolution melting analysis (HRMA) and next-generation sequencing (NGS), offer deeper insight into methylation profiles, though they come with higher costs and longer turnaround times [44]. One of the most famous multi-cancer early detection test focusing on ctDNA methylation signatures to detect multiple cancers is the Grail Galleri. With one large study suggesting the potential to identify over 50 tumor types from a single blood draw [45], its performance specifically in endometrial, cervical, or ovarian cancer still requires further validation and FDA approval is yet to come.
- Because ctDNA reflects both genetic and epigenetic aspects of a tumor, measuring it can guide targeted therapy or detect minimal residual disease [46]. Techniques like droplet digital PCR (ddPCR) can reliably identify mutations (e.g., TP53, KRAS) with over 99% specificity. NGS-based panels also allow for simultaneous evaluation of multiple alterations. Importantly, multi-analyte tests now integrate ctDNA with additional biomarkers, such as selected proteins, combined with machine-learning algorithms to achieve early multi-cancer detection. For instance, CancerSEEK includes 16 gene mutations and 8 circulating proteins, and has reported sensitivities exceeding 70–90% for certain tumor types [46, 47]. In gynecologic oncology, ctDNA monitoring has shown practical clinical applications; for instance, Toboni et al. demonstrated in advanced or recurrent endometrial cancer that ctDNA status, measured via the Signatera™ multiplex PCR assay, correlates with clinical outcomes [48]. Personalized NGS approaches have likewise been used to measure responses to PARP inhibitors in ovarian cancer. Large-scale sequencing of ctDNA has further delineated actionable molecular profiles in advanced endometrial cancer [49]. Despite these advances, ctDNA test sensitivity can decrease when ctDNA levels are extremely low, emphasizing the necessity of high-quality plasma samples and rigorous laboratory protocols [50]. Several ctDNA-based assays have received FDA approval, including Guardant360 and FoundationOne® Liquid CDx [44]. FoundationOne® Liquid has also been applied to endometrial and other advanced tumors (lung, colon, ovarian, breast), where ctDNA fraction guides real-time management [51]. Moreover, low-coverage Illumina HiSeq2500 sequencing studies indicate that cfDNA can provide relevant molecular insights even without direct tumor references, further extending its diagnostic and monitoring utility [51].
- These small, stable, tissue-specific RNA molecules continue to generate interest in clinical oncology. They can be measured by qPCR or microarray for targeted screening, while NGS allows a more comprehensive miRNA survey [52]. Exosomal miRNAs in particular reflect ongoing tumor activity, but isolation protocols significantly affect yield and data quality. For example, ultracentrifugation can retrieve plentiful exosomes but risks vesicle damage, whereas size-exclusion chromatography (SEC) better preserves vesicle integrity but may be labor-intensive [52]. Microfluidic platforms that integrate both size-based and immunoaffinity-based isolation are emerging as a reliable alternative with promising specificity and recovery [53]. Furthermore, new techniques like ELSA-seq (Enhanced Linear-Splinter Amplification Sequencing) have shown predictive accuracies up to 97% in ovarian cancer, underscoring the role of ctDNA methylation profiling [54].

### Biomarkers could reveal the story of gynecological cancers

Each biomarker type, ctDNA, CTCs, cfRNA, TEPs, and exosomes, uniquely reflects a tumor’s genetic profile, proliferative behavior, and metastatic potential.

- ctDNA: Tracing Tumor Genetic Footprints.
  Because ctDNA captures the active genetic alterations that drive a patient’s cancer, it can detect minimal residual disease (MRD) even before clinical or imaging signs emerge [55]. In ovarian cancer, clinicians often track ctDNA levels to forecast outcomes and tailor therapies, whereas in endometrial carcinoma, fluctuating ctDNA can suggest a heightened recurrence risk, prompting closer monitoring or additional intervention.
- CTCs: Migratory Messengers of Tumors.
  Unlike ctDNA, which reflects genetic mutations, CTCs are intact cancer cells that escape from primary or metastatic lesions [46, 56, 57]. Detecting even a small number can expose a latent potential for spread. In ovarian cancer, CTC enumeration and molecular profiling reveal micrometastatic disease, while in endometrial cancer, the presence of CTCs, sometimes detectable in early stages, may signal impending recurrence [58–61]. A notable hurdle is that CTCs can undergo epithelial-to-mesenchymal transition (EMT), losing conventional surface markers and thereby requiring more advanced technologies for reliable capture.
- cfRNA and TEPs: Unveiling Subtler Layers of Tumor Biology.
  Not all tumor-related signals come from DNA fragments or whole cells. cfRNA (including mRNA, miRNA, circRNA, and lncRNA) reflects active gene expression in tumors and can remain surprisingly stable in circulation [55]. In ovarian cancer, certain miRNAs and circRNAs appear promising for diagnosing early-stage disease, and preliminary research on mRNA within utero-tubal lavage fluid hints at additional detection approaches. Beyond cfRNA, TEPs offer another vantage point; platelets “educated” by cancer cells incorporate malignant RNA signatures that can distinguish tumors from benign states, though more validation is needed before such tests become routine.
- Exosomes: Tiny Couriers of Tumor Cargo.
  Exosomes encapsulate a tumor’s molecular fingerprint, making them promising candidates for noninvasive diagnosis, prognosis, and follow-up [55]. For example, in cervical cancer, exosomal circSLC26A4 correlates with advanced FIGO stages and lymph node metastases, suggesting its utility in gauging disease severity [62]. Exosome-based assays also show potential in differentiating benign from malignant ovarian masses, though further clinical trials are required to standardize their application.

### Does chemotherapy have an impact on liquid biopsy detection rate?

Chemotherapy can alter both the release and detectability of ctDNA and CTCs. Early in treatment, tumor cell apoptosis and necrosis often lead to short-term spikes in ctDNA as dying cells shed DNA fragments [63]. Although this increase may seem concerning, it typically signifies a positive treatment response rather than disease progression. Over time, stable declines in ctDNA or CTC counts indicate meaningful tumor regression [64], whereas persistently elevated or resurgent biomarker levels suggest drug resistance or minimal residual disease [65]. For instance, platinum-resistant ovarian tumors generally exhibit higher CTC counts [65], while increasing ctDNA during PARP inhibitor therapy can signal emerging treatment failure [66, 67]. Epigenetic modifications, like HOXA9 promoter methylation, further refine therapeutic choices and prognostication [68]. By serially monitoring these biomarkers, clinicians can distinguish between chemotherapy-induced cell death and emerging resistance [69].

### Ideal Scenarios for Liquid Biopsy: What are the clear-cut situations or clinical indications in gynecological cancers where liquid biopsy is particularly advantageous?

Liquid biopsy is especially beneficial in gynecological cancers for early detection, often identifying malignancies before symptoms appear, particularly in high-risk groups [70]. It also facilitates real-time monitoring of therapeutic responses, enabling treatment modifications if ctDNA levels rise or remain elevated. Post-treatment detection of minimal residual disease (MRD) via ctDNA can guide follow-up and additional interventions. In addition, liquid biopsy pinpoints actionable mutations for personalized therapy and reveals tumor heterogeneity to refine treatment plan. Finally, routine ctDNA assessments can determine recurrence risk, ensuring targeted surveillance for high risk patients [71].

### Warnings on liquid biopsy

Liquid biopsy can be unreliable under certain biological and technical conditions. In early-stage (I or II) disease, ctDNA concentrations can be more than tenfold lower than in advanced disease, greatly increasing the likelihood of false negatives [72]. Additionally, ctDNA’s short half-life, ranging from 15 minutes to 2 hours, makes it susceptible to degradation, while cell lysis can further contaminate samples unless handled swiftly under stringent protocols [73, 74]. Exosomes pose similar hurdles: tumor-derived vesicles often account for less than 2% of circulating exosomes and are rapidly cleared, requiring high-throughput, highly sensitive methods for accurate analysis [75, 76]. Although TEPs are abundant and simple to isolate, they may capture only a fraction of a tumor’s genetic profile [72]. Combined, these issues can lead to suboptimal or false-negative outcomes, particularly in heterogeneous tumors or when specific histopathological detail is essential for treatment decisions [77]. Understanding and compensating for such biological complexities is crucial to preserving the accuracy and clinical utility of liquid biopsy results.

### False positives and negatives in liquid biopsy

Biological complexity and technical challenges often lead to false results in liquid biopsies for gynecological cancers. For instance, Fourier transform infrared (FTIR) spectroscopy can reach nearly 99% sensitivity, but at the cost of lowered specificity, causing benign conditions to appear malignant [78] . Conversely, boosting specificity risks missing early tumors that release minimal ctDNA [78]. In fluorescence liquid biopsy protocols, benign ovarian cysts may mimic malignant fluorescence signals, raising false positives, while some ovarian tumors lack a distinct fluorescence shift, increasing false negatives [79]. Similarly, in microvesicle proteomics of uterine fluid, benign conditions can mimic malignant signatures, leading to over-diagnosis, while low microvesicle counts in early-stage cancer may go undetected [77].

### Emerging biotechnological techniques under investigation for liquid biopsy

A variety of next-generation laboratory methods promise to bolster biomarker detection and revolutionize current diagnostic workflows. Digital PCR partitions samples to precisely count nucleic acids, obviating reference standards and enhancing sensitivity for rare ctDNA mutations [80]. NGS provides deep genomic snapshots, capturing tumor heterogeneity and enabling real-time monitoring of emerging subclones [81]. Nanoparticle-based assays detect signals from low-abundance targets and can be integrated into portable, point-of-care devices [82]. Meanwhile, CRISPR-Cas systems leverage genome-editing principles for rapid, targeted nucleic-acid detection, cutting turnaround times and facilitating flexible assay design [83]. Single-cell sequencing dissects individual CTCs at the genomic and epigenomic levels, identifying subtle subclones that fuel resistance or relapse [84]. Finally, electrochemical and optical biosensors transform biological interactions into quantifiable signals, delivering cost-effective, sensitive detection of biomarkers such as miRNAs, ctDNA, and exosomes [85].

### AI Integration in Liquid Biopsy

Artificial intelligence can substantially enhance liquid biopsy applications by synthesizing multiple layers of cfDNA fragmentomics, methylomics, and epigenetic data, thereby boosting both sensitivity and specificity. Combining fragmentation-focused approaches like DELFI with three-dimensional genome mapping tools pinpoints tumor-specific changes in cfDNA length and distribution while determining tissue of origin via chromatin conformation and nucleosome positioning [33, 86]. Further metrics, such as promoter fragmentation entropy and windowed protection scores, illuminate gene activation and transcription factor binding [87, 88]. Orientation-aware fragmentation refines tissue-of-origin analyses by mapping nucleosome placement in open chromatin areas [89]. Methylation-based methods classify cfDNA fragments based on inferred methylation patterns, attributing them to specific tissues [90, 91]. Different databases like consolidate broad omics data with cfDNA sequencing, offering a holistic perspective on gene regulation and fragmentomics [92].

### Liquid biopsy in ovarian cancer

- Screening
  Liquid biopsy screening is particularly relevant for BRCA1/2 mutation carriers, whose ovarian cancer risk is two to four times higher [93]. Various biomarkers have shown promise. Plasma protein signatures (e.g., SPARC, THBS1) have been proposed [94], and uterine lavage analyses identified a seven-protein panel with over 99% negative predictive value [95]. Pap test–based methods demonstrated 52% overall accuracy and 26% sensitivity for eight-gene panels [96], while p53 variants were found in archival Pap smears up to six years before diagnosis [97]. Methylation profiling of tumor suppressor genes ranges from 41% to 100% sensitivity, with OPCML emerging as a particularly robust biomarker [98].
- Early Diagnosis
  Meta-analysis of circulating cell-free DNA demonstrated 70% sensitivity and 90% specificity, with a diagnostic odds ratio of 26.05 and negative likelihood ratio of 0.34 [99]. Updated meta-analyses incorporating 22 studies confirmed these findings with slightly improved pooled sensitivity of 73% while maintaining 90% specificity [100]. miRNA analysis showed strong diagnostic potential, with meta-analyses revealing 89% sensitivity and 64% specificity. Multiple miRNA panels demonstrate superior performance compared to single markers, with diagnostic odds ratios of 30.06 versus 13.21 [101]. Recent research has identified nine upregulated miRNAs in ovarian cancer patients, with MiR-145 and MiR-205 showing the highest fold change exceeding 2-fold [102]. Studies have consistently demonstrated improved accuracy when combining CA125 with investigated miRNAs compared to either marker alone.
- Diagnosis
  ctDNA correlates well with tumor DNA, though heterogeneity may affect accuracy [103]. Extracellular vesicles containing miRNAs also display notable differences in expression between ovarian cancer patients and controls [104]. Diagnostic performance varies according to plasma vs. serum collection, extraction protocols, hormonal factors, and menstrual status [105, 106].
- Staging
  Several liquid biopsy biomarkers help distinguish disease stages. For instance, CD117 expression on cells and on extracellular vesicles is significantly higher in recurrent disease, particularly in high-grade serous carcinoma [107]. Advanced-stage ovarian cancer typically features elevated cfDNA levels [108] and higher ctDNA detection rates [109]. Low-coverage whole-genome sequencing of plasma cfDNA can differentiate early- from late-stage cancers [110]. Exosomal miR-205 and certain metabolic markers (phenylpyruvic acid, 4-hydroxyphenylpyruvic acid) are also linked to advanced disease [55, 111].
- Evaluation for Treatment Response
  Liquid biopsy enables real-time monitoring of chemotherapy and PARP inhibitor responses [55]. ctDNA tracks primary and acquired resistance; for instance, in BRCA-mutated cases, increased HOXA9 methylation during treatment correlates with poor PARP inhibitor efficacy [68]. Serum soluble PD-L2 levels can predict platinum response since high levels demonstrate association with platinum therapy response and low levels indicate resistance and poorer prognosis [112]. For high-grade serous ovarian cancer patients, ctDNA analysis detects chemotherapy response earlier than CA-125, with TP53 mutant allele fraction serving as a predictor of poor outcomes and rapid progression [113]. Extracellular vesicle markers CD24 and EpCAM in plasma show elevated levels in non-responding versus responding patients [114]. Additionally, cfDNA analysis through next-generation sequencing before and during treatment can track tumor progression and genetic evolution during chemotherapy [115].
- Follow-up
  For post-treatment surveillance, ctDNA shows high prognostic utility. In high-grade serous ovarian cancer, elevated ctDNA (≥0.2 copies/μL) at three months post-chemotherapy correlates with a 58.3% recurrence risk compared to patients with low levels (<0.2 copies/μL), who demonstrate only 6.7% recurrence risk [63]. Liquid biopsy can detect relapse up to seven months earlier than CT imaging [116] and outperforms CA125 in predicting progression [117]. Elevated ctDNA levels are also associated with worse survival, and HOXA9 methylation positivity raises the relapse risk more than threefold [118]. Additional studies have confirmed that ctDNA quantification can indicate recurrence months before conventional clinical methods, providing an objective definition of complete cytoreduction [119].

### Liquid biopsy in endometrial cancer

- Screening
  Endometrial cancer (EC) often presents asymptomatically, prompting interest in minimally invasive screening. A major systematic review reported 56 blood-based biomarker studies and only one employing urine [120]. Recent methods emphasize site-specific sampling to improve sensitivity and specificity. A tampon-based test evaluating methylated DNA markers in vaginal fluid reached 76% sensitivity and 96% specificity (AUC=0.88) in 192 participants, and further refinements raised sensitivity to 82% [121]. Another technique, endometrial fluid sampling akin to saline infusion sonohysterography, revealed distinct microRNAs (miR-183-5p, miR-429, miR-146a-5p) differentiating malignant from benign cases in both an exploratory and validation cohort [122]. Although these strategies reduce nonspecific signals by sampling near the tumor site, large-scale validation remains necessary [120–122].
- Diagnosis
  While standard endometrial biopsy remains the gold standard, liquid biopsy may aid patients who cannot undergo invasive sampling or who yield inadequate tissue. Exosomal, proteomic, and metabolomic markers, along with ctDNA, cfDNA, and survivin-expressing cells, have all shown promise but have not yet matched the accuracy of tissue biopsy [120]. Nonetheless, certain exosomal microRNAs (e.g., miR-21, miR-27a, miR-223) and proteomic markers (YKL-40, DJ-1) demonstrated AUC values approaching 0.925 [120]. In a pilot study, CTCs were detectable in 80% of ovarian venous blood but absent in peripheral blood of patients with early-stage EC, suggesting a localized dissemination route [58]. Meanwhile, cfDNA profiling in 61 advanced EC patients had 87.5% concordance with tissue-based molecular classification and uncovered actionable alterations in 65% [123]. Another methodological study combined suction curettage without anesthesia, liquid-based cytology, and micro-histology to achieve 92.3% sensitivity and 100% specificity in 100 patients [122]. Collectively, these findings indicate that blood, uterine aspirates, and other minimally invasive samples, together with molecular profiling, may guide targeted EC therapies [58, 120, 122, 123].
- Follow-up and Prognostic Assessment
  Liquid biopsy also aids in tracking disease progression and refining prognosis. In a prospective study of 198 patients, 29.38% had detectable ctDNA at surgery; this correlated with higher tumor grade and advanced stage [124]. Elevated cfDNA (>25 ng/mL) was linked to shorter DFS and DSS, and ctDNA positivity detected relapse around 4.7 months before clinical or radiologic confirmation [124]. Another pilot comparing ctDNA and tumor DNA in 21 patients found shared mutations in two-thirds of cases, although the detection of DNMT3A and TET2 mutations in older individuals emphasized the confounding issue of clonal hematopoiesis [59]. Microsatellite instability (MSI) monitored via ddPCR in 90 uterine aspirates showed 96.67% concordance with mismatch repair protein status; in one case, MSI markers identified recurrence before clinical detection [124]. Despite these advances, about 20% of relapse cases were ctDNA-negative, highlighting the need for assay improvements or multipronged biomarker approaches [124, 125].
- Perspectives and Emerging Techniques
  Ongoing refinements aim to enhance liquid biopsy’s sensitivity, specificity, and practicality. An exosome metabolic fingerprinting study that used a Fe3O4@COF@Au-Apt nanoplatform evaluated 105 plasma samples (51 EC, 54 controls) and attained an AUC of 0.924 in blind testing [126]. Machine learning identified four metabolites, hydroxychalcone, L-acetylcarnitine, elaidic acid, and glutathione, that produced 94.9% classification accuracy [126]. Similar AI-assisted analytics, along with advanced sampling (e.g., suction curettage, tampon-based fluid collection) and molecular assays (NGS, ddPCR-MSI), could further transform EC detection and monitoring [120, 121, 126].

### Liquid Biopsy in Cervical Cancer

- Early Diagnosis and Screening
  Human papillomavirus (HPV) is the primary etiologic agent in cervical cancer, with subtypes 16 and 18 accounting for about 70% of cases [127]. Digital droplet PCR of HPV-specific genes E7 and L1 in plasma samples from 138 Hong Kong Chinese women with cervical cancer revealed that higher viral loads correlate with increased five-year recurrence and mortality risk, highlighting circulating HPV DNA as a critical surveillance marker [128].
- Diagnosis and Disease Monitoring
  Several serum protein markers offer clinical utility in monitoring cervical cancer. For example, VCAM-1 and ICAM-1 levels can predict radiotherapy or chemoradiotherapy response in a cohort of 189 patients [129]. Regulatory proteins Rspo1 and Slit2 correlate positively with radiotherapy tolerance and negatively with hematologic and cardiac toxicity [130]. Pre-treatment hemoglobin below 11 g/dl is linked to treatment resistance, whereas hemoglobin above 12.7 g/dl is significantly associated with complete radiotherapy response (p<0.001), as well as improved overall and disease-free survival [131].
- Staging and Treatment Response Evaluation
  Multiple studies have established threshold values for Squamous cell carcinoma antigen (SCC-Ag) across clinical contexts. Pre-treatment levels above 2 ng/ml predict distant recurrence within five years [132], while levels exceeding 6.5 ng/ml suggest a benefit from adjuvant chemotherapy, reducing systemic recurrence [133]. Post-treatment thresholds above 1.15 ng/ml correlate with decreased three-year overall survival (84%) in chemoradiotherapy patients, and levels exceeding 1.20 ng/ml predict worse survival (95%) in radiotherapy-only cohorts. Additionally, post-treatment SCC-Ag above 1.0 ng/ml is linked to higher recurrence risk in stage IB–IIIB disease [134, 135]. Analyzing cfDNA in 93 plasma samples from 57 patients demonstrated a marked decrease in allele fraction deviation (AFD) after treatment (p=0.029), correlating cfDNA reduction with tumor shrinkage and confirming the predictive value of AFD for disease progression and relapse [136].
- Follow-up and Surveillance
  In a study of 99 patients with locally advanced cervical cancer (FIGO stage IIB–IVA), elevated CTCs and SCC-Ag levels emerged as independent prognostic factors for disease-free survival. A combined CTC/SCC-Ag risk model outperformed individual markers, and multivariate analysis confirmed serum CTC count, FIGO stage, and serum SCC-Ag as independent predictors for two-year disease-free survival [137]. Monitoring treatment response through VEGF1 reduction, alongside tissue HIF-1a levels, correlated strongly with complete chemoradiotherapy response [138]. Implementing cfDNA surveillance enabled early detection of treatment response and progression, while serial plasma sampling facilitated longitudinal genomic tracking. Systematic examination of circulating HPV DNA similarly reflected disease status, with shifts in viral load providing an early sign of therapy response and recurrence risk [139]. Integrating multiple biomarkers yields superior clinical utility over single-marker approaches, particularly in evaluating early response, anticipating recurrence, adjusting treatment strategies, and ensuring long-term surveillance.

### Analogistic practical considerations: liquid biopsy versus pathological analysis

Empirical data from a broad range of tumor types underscore how liquid biopsy has become powerful, minimally invasive tool that complements traditional tissue biopsy, expanding the horizons of precision oncology (Table 3). Although many of these studies center on non-gynecological cancers, they provide instructive analogies for potential applications in endometrial, cervical, and ovarian tumors. Capturing tumor heterogeneity in real time provides vital insights into mutational burdens, emerging resistance, and minimal residual disease [140]. However, important challenges persist. Early-stage malignancies may release scant amounts of ctDNA, limiting sensitivity and increasing the risk of false negatives, and clonal hematopoiesis can obscure ctDNA findings in older patients [141, 142]. Further, while liquid biopsy excels at providing a dynamic portrait of disease evolution, it lacks the exhaustive morphological data gleaned from tissue specimens, which remain the gold standard for grading, staging, and comprehensive immunohistochemical assessments [143, 144]. Despite these constraints, the real-time feedback offered by liquid biopsy is invaluable for guiding therapy modifications, detecting emerging driver mutations, and flagging early relapse, often before radiographic imaging can confirm disease progression [145, 146]. Moreover, it can be particularly advantageous when tumor sites are anatomically difficult to biopsy or when patients’ overall health precludes surgical intervention [147]. Current investigative trends include refining molecular assays for higher sensitivity in early-stage tumors and exploring additional biofluids, such as ascites and cerebrospinal fluid, to expand diagnostic reach [148]. In moving forward, the synergy between liquid biopsy and traditional pathological analysis promises the most comprehensive patient stratification. Tissue biopsy remains indispensable for initial diagnosis, detailed morphological evaluation, and robust molecular classification; meanwhile, liquid biopsy offers a dynamic and repeatable snapshot of tumor evolution that optimizes personalized treatment and surveillance strategies [141, 142]. By integrating both modalities, clinicians can capitalize on real-time genetic insights while retaining the diagnostic depth essential for accurate staging and targeted therapy. Ultimately, ongoing technological advances and interdisciplinary collaboration will further refine these approaches, facilitating earlier detection, improved outcomes, and a more patient-centric model of cancer care.

**Table 3:**
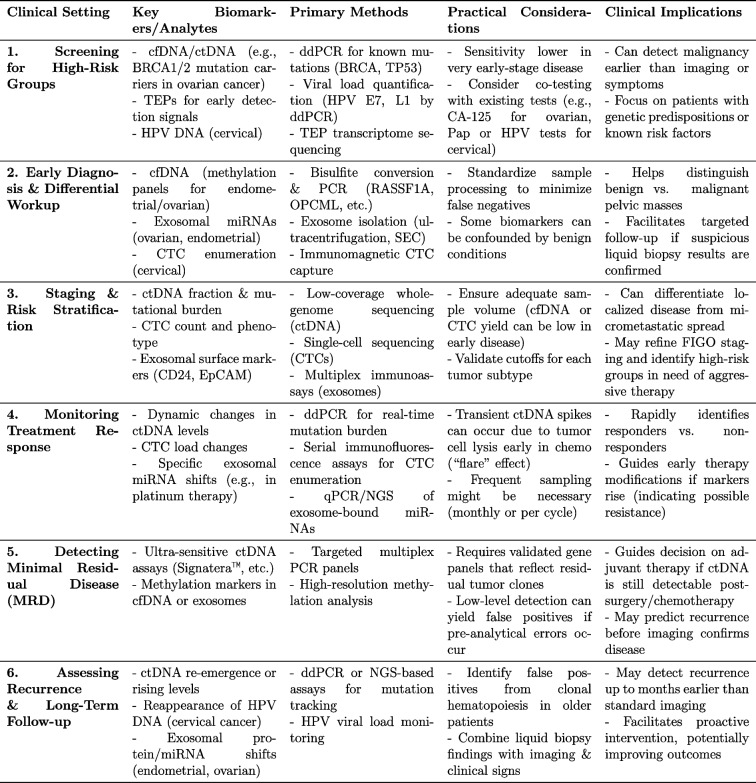
Clinical and practical overview of liquid biopsy use across gynecological oncology

## Conclusion

Liquid biopsy has emerged as a pivotal, noninvasive tool for the detection and monitoring of gynecological cancers, offering real-time insights into tumor biology that complement standard tissue-based approaches, but it has not yet been entered into routine clinical practice in gynecological oncology. This review aims to clarify the fundamental biological rationale and technical foundations of various liquid biopsy biomarkers in gynecological oncology, which is essential for both researchers and clinicians seeking to contextualize current evidence. Indeed, while many studies report encouraging performance in controlled or advanced disease settings, true clinical utility remains unproven in larger, prospective trials. A further consideration is the growing interest in multi-cancer early detection tests that are still awaiting FDA approval [149, 150]. Although these platforms have generated considerable excitement, real-world data has highlighted potential shortcomings. Sensitivity for early stage disease can be modest, raising questions about whether early detection of a tumor will ultimately translate into improved survival or merely reflect lead-time bias. Likewise, even a small false-positive rate, when projected to a population-wide screening program, could lead to unnecessary, invasive diagnostic procedures and substantial costs. For gynecological malignancies, specifically, data remain sparse, underscoring the need for robust clinical trials that assess actual reductions in cancer-specific mortality rather than rely solely on stage-shift endpoints. By contrasting robust technical advances with these variables, and sometimes limited, clinical outcomes, we aim to underscore that liquid biopsy, though innovative, is not a one-size-fits-all solution; its utility must be assessed in a disease- and context-specific manner. By outlining the mechanistic basis, we illustrate where current approaches might fail under practical conditions and how targeted technical improvements could address these pitfalls. Ultimately, ongoing interdisciplinary efforts, larger prospective trials, cost-effectiveness analyses, and meticulous follow-up will be essential for resolving these gaps and ensuring that promising laboratory data translate into meaningful, patient-centered outcomes in gynecologic oncology.

## Data Availability

No datasets were generated or analysed during the current study.
