# Correction: Liquid biopsy in gynecological cancers: a translational framework from molecular insights to precision oncology and clinical practice

**DOI:** 10.1186/s13046-025-03429-0

**Published:** 2025-05-30

**Authors:** Canio Martinelli, Alfredo Ercoli, Giuseppe Vizzielli, Sharon Raffaella Burk, Maria Cuomo, Vrunda Satasiya, Housem Kacem, Simone Braccia, Giulio Mazzarotti, Irene Miriello, Manuela Nana Tchamou, Stefano Restaino, Martina Arcieri, Alice Poli, Veronica Tius, Silvana Parisi, Stefano Pergolizzi, Giuseppe Iatì, Chiara Conti Nibali, Cristina Pizzimenti, Ludovica Pepe, Antonio Ieni, Salvatore Cortellino, Antonio Giordano

**Affiliations:** 1 Sbarro Institute for Cancer Research and Molecular Medicine and Center of Biotechnology, College of Science and Technology, Temple University, 1900 N 12 St, Philadelphia, PA 19122 USA; 2 Department of Human Pathology of Adult and Childhood “Gaetano Barresi”, Unit of Obstetrics and Gynecology, University of Messina, Via Consolare Valeria 1, Messina, 98124 Italy; 3 Clinic of Obstetrics and Gynecology, Santa Maria Della Misericordia” University Hospital, Azienda Sanitaria Universitaria Friuli Centrale, Udine, Italy; 4 Department of Medical Biotechnology, University of Siena, Via Aldo Moro 2, Siena, 53100 Italy; 5 Department of Life, Health and Environmental Sciences, University of L’Aquila, L’Aquila, Italy; 6 Department of Pharmacy, School of Medicine, University of Naples Federico II, Via Domenico Montesano 49, Naples, 80131 Italy; 7 Radiation Oncology Unit, Department of Biomedical, Dental Science and Morphological and Functional Images, University of Messina, Messina, 98125 Italy; 8 Section of Pathological Anatomy, Department of Human Pathology of Adult and Evolutive Age “Gaetano Barresi”, G. Martino Hospital, Messina, 98125 Italy; 9 Clinical and Translational Oncology, Scuola Superiore Meridionale (SSM), Naples, Italy; 10 Laboratory of Molecular Oncology, Research Hospital, Campobasso, 86100 Italy; 11 SHRO Italia Foundation ETS, Candiolo, Turin, Italy

Correction: *J Exp Clin Cancer Res***44**, 140 (2025)

10.1186/s13046-025-03371-1

Following the publication of the original article [1], the authors identified minor errors in the body of the article. The corrections are highlighted in bold.

**Table:** 

Error	Correction
The detection of these markers has the potential to revolutionize cancer management by enabling earlier detection, providing novel data to personalize treatments, and predicting disease recurrence before clinical imaging **and predicting disease recurrence before clinical** **imaging** can confirm progression, thereby also guiding complex clinical decision-making.	The detection of these markers has the potential to revolutionize cancer management by enabling earlier detection, providing novel data to personalize treatments, and predicting disease recurrence before clinical imaging can confirm progression, thereby also guiding complex clinical decision-making.
**Their** attractive biomarkers for various gynecological malignancies	They are attractive biomarkers for various gynecological malignancies.
NGS-based panels also allow simultaneous evaluation of multiple alterations.	NGS-based panels also allow **for** simultaneous evaluation of multiple alterations.
Importantly, multi-analyte tests now integrate ctDNA with additional biomarkers, such as selected proteins, combined with machine-learning algorithms to achieve multi-cancer **early** detection.	Importantly, multi-analyte tests now integrate ctDNA with additional biomarkers, such as selected proteins, combined with machine-learning algorithms to achieve **early** multi-cancer detection.
In ovarian cancer, CTC enumeration and molecular profiling **unveil** micrometastatic disease, while in endometrial cancer,	In ovarian cancer, CTC enumeration and molecular profiling reveal micrometastatic disease, while in endometrial cancer,
Exosomes encapsulate a tumor’s molecular fingerprint, making them promising for noninvasive diagnosis, prognosis, and follow-up	Exosomes encapsulate a tumor’s molecular fingerprint, making them promising **candidates** for noninvasive diagnosis, prognosis, and follow-up
For example, in cervical cancer, exosomal circSLC26A4 correlates with advanced FIGO stages and lymph node metastases, suggesting utility in gauging disease severity	For example, in cervical cancer, exosomal circSLC26A4 correlates with advanced FIGO stages and lymph node metastases, suggesting **its** utility in gauging disease severity
Although this **rise** may seem concerning,	Although this increase may seem concerning,
Post-treatment, **detecting** minimal residual disease (MRD) via ctDNA can guide follow-up and additional interventions.	Post-treatment detection of minimal residual disease (MRD) via ctDNA can guide follow-up and additional interventions.
Finally, routine ctDNA assessments can determine recurrence risk, ensuring targeted surveillance for **patients at high risk**	Finally, routine ctDNA assessments can determine recurrence risk, ensuring targeted surveillance for high risk patients
Understanding and compensating for such biological complexities is crucial to **preserve** the accuracy and clinical utility of liquid biopsy results.	Understanding and compensating for such biological complexities is crucial to preserving the accuracy and clinical utility of liquid biopsy results.
Biological complexity and technical challenges often **drive** false results in liquid biopsies for gynecological cancers.	Biological complexity and technical challenges often lead to false results in liquid biopsies for gynecological cancers.
**Nanoparticlebased** assays signals from low-abundance targets	Nanoparticle-based assays **detect** signals from low-abundance targets
Methylation-based methods classify cfDNA fragments **by** inferred methylation patterns, attributing them to specific tissues [90, 91].	Methylation-based methods classify cfDNA fragments based on inferred methylation patterns, attributing them to specific tissues [90, 91].
Meta-analysis of circulating cell-free DNA demonstrates 70% sensitivity and 90% specificity, with a diagnostic odds ratio of 26.05 and negative likelihood ratio of 0.34 [99]. Updated meta-analyses incorporating 22 studies **confirm** these findings with slightly improved pooled sensitivity of 73% while maintaining 90% specificity [100]. **Micro-** **RNA** analysis **shows** strong diagnostic potential, with meta-analyses revealing 89% sensitivity and 64% specificity. Multiple miRNA panels demonstrate superior performance compared to single markers, with diagnostic odds ratios of 30.06 versus 13.21 [101].	Meta-analysis of circulating cell-free DNA demonstrated 70% sensitivity and 90% specificity, with a diagnostic odds ratio of 26.05 and negative likelihood ratio of 0.34 [99]. Updated meta-analyses incorporating 22 studies confirmed these findings with slightly improved pooled sensitivity of 73% while maintaining 90% specificity [100]. miRNA analysis showed strong diagnostic potential, with meta-analyses revealing 89% sensitivity and 64% specificity. Multiple miRNA panels demonstrate superior performance compared to single markers, with diagnostic odds ratios of 30.06 versus 13.21 [101].
Studies consistently **demonstrate** improved accuracy when combining CA125 with investigated miRNAs compared to either marker alone.	Studies have consistently demonstrated improved accuracy when combining CA125 with investigated miRNAs compared to either marker alone.
Extracellular vesicles containing miRNAs also display notable expression differences between ovarian cancer patients and controls [104].	Extracellular vesicles containing miRNAs also display notable **differences** in expression between ovarian cancer patients and controls [104].
Diagnostic performance varies **by** plasma vs. serum collection, extraction protocols, hormonal factors, and menstrual status	Diagnostic performance varies according to plasma vs. serum collection, extraction protocols, hormonal factors, and menstrual status
Liquid biopsy can detect relapse up to seven months earlier than CT imaging [116]. **and** outperforms CA125 in predicting progression [117].	Liquid biopsy can detect relapse up to seven months earlier than CT imaging [116] and outperforms CA125 in predicting progression [117].
Elevated ctDNA levels also **associate** with worse survival, and HOXA9 methylation positivity raises the relapse risk more than threefold [118]. Additional studies confirm that ctDNA quantification can indicate recurrence months before conventional clinical methods, providing an objective definition of complete cytoreduction [119].	Elevated ctDNA levels are also associated with worse survival, and HOXA9 methylation positivity raises the relapse risk more than threefold [118]. Additional studies have confirmed that ctDNA quantification can indicate recurrence months before conventional clinical methods, providing an objective definition of complete cytoreduction [119].
In a pilot study, CTCs were detectable in 80% of ovarian venous blood but absent in peripheral blood among early-stage EC patients, suggesting a localized dissemination route [58].	In a pilot study, CTCs were detectable in 80% of ovarian venous blood but absent in peripheral blood **of patients with** early-stage EC, suggesting a localized dissemination route [58].
**By** capturing tumor heterogeneity in real time provides vital insights	Capturing tumor heterogeneity in real time provides vital insights
Despite these constraints, the real-time feedback that liquid biopsy offers is invaluable for guiding therapy modifications, detecting emerging driver mutations, and flagging early relapse, often before radiographic imaging can confirm disease progression [145, 146].	Despite these constraints, the real-time feedback **offered by** liquid biopsy is invaluable for guiding therapy modifications, detecting emerging driver mutations, and flagging early relapse, often before radiographic imaging can confirm disease progression [145, 146].
A further consideration is the growing interest in multi-cancer early detection tests that are still waiting **for** FDA approval [149, 150]. Although these platforms have generated considerable excitement, real-world data highlight potential shortcomings. Sensitivity for earlystage disease can be modest, raising questions about whether finding a tumor earlier will ultimately translate into improved survival or merely reflect lead-time bias.	A further consideration is the growing interest in multi-cancer early detection tests that are still awaiting FDA approval [149, 150]. Although these platforms have generated considerable excitement, real-world data has highlighted potential shortcomings. Sensitivity for early stage disease can be modest, raising questions about whether early detection of a tumor will ultimately translate into improved survival or merely reflect lead-time bias.
…and ensuring that promising laboratory data translate into meaningful, patient-centered outcomes in gynecological. **oncology.**	…and ensuring that promising laboratory data translate into meaningful, patient-centered outcomes in gynecologic oncology.

The corrections do not compromise the validity of the conclusions and the overall content of the article. The original article [1] has been updated.
